# Robot-assisted laparoscopic augmentation ileocystoplasty for neurogenic bladder: case report and structured literature review

**DOI:** 10.3389/fsurg.2026.1767111

**Published:** 2026-10-22

**Authors:** Jia You, Cheng Lan, Xu-fei Duan, Hai-tao Chen

**Affiliations:** 1 Department of Pediatric Surgery, Wuhan Children’s Hospital (Wuhan Maternal and Child Healthcare Hospital), Tongji Medical College, Huazhong University of Science and Technology, Wuhan, China; 2 Department of Neurology, The Central Hospital of Wuhan, Tongji Medical College, Huazhong University of Science and Technology, Wuhan, China

**Keywords:** augmentation ileocystoplasty, minimally invasive surgery, mitrofanoff procedure, neurogenic bladder, robotic surgery, urological reconstruction, structured literature review, pooled analysis

## Abstract

**Background:**

Bladder augmentation is generally reserved for selected patients failing conservative management or with unsafe storage pressures/compliance for neurogenic bladder (NGB) patients. The robotic-assisted laparoscopic approach for urinary issues has gained attention. This study evaluates the feasibility and safety of robot-assisted laparoscopic augmentation ileocystoplasty (RALAI) in treating NGB.

**Methods:**

We present a case study of an 8.7-year-old male patient diagnosed with NGB, characterized by a preoperative bladder capacity of 198 ml, compliance of 13.6 ml/cm H_2_O, maximum detrusor pressure of 24 cm H_2_O, and a post-void residual volume of 250 ml. The patient underwent RALAI with the creation of a Mitrofanoff conduit. Subsequently, we conducted a comprehensive literature review on RALAI for NGB patients, covering the period from January 1, 2004, to December 31, 2024, utilizing databases such as PubMed, Web of Science, China National Knowledge Infrastructure (CNKI), and Wanfang. The search employed keywords including “neurogenic bladder,” “ileocystoplasty,” and “robot.”

**Measurements:**

The primary objective was to ascertain the surgical success rate of RALAI without conversion to open surgery. Secondary objectives included assessing complications within 90 days postoperatively, such as stomal complications, intestinal issues, and infections.

**Results:**

The RALAI procedure was completed without conversion to open surgery in 86/92 patients (93.5%, 95% CI 88.4–98.5%), with a conversion rate of 6.5% (95% CI 1.6–11.4%). No intraoperative complications (e.g., visceral injury, hemorrhage or anesthetic events) directly attributable to the surgical technique were recorded). In the pooled cohort of 92 patients, the RALAI procedure demonstrated favorable patient-centred outcomes: postoperative bladder capacity increased substantially in the majority of reported cases; the rate of early postoperative complications was 38.0% (95% CI 28.1–48.0%), with 13.0% (95% CI 6.2–19.9%) requiring secondary surgical revision. Urinary tract infections occurred in 14.1% of patients. In our own index case, the patient achieved bladder capacity of 380 ml and social continence, resumed intermittent catheterization and spontaneous voiding, and remained free of urinary tract infections (UTIs) and bladder stones at 3 years.

**Conclusions:**

Our findings indicate that RALAI is a feasible minimally invasive option for selected NGB patients, with a conversion rate of approximately 6.5%. However, the true value of the procedure should be assessed by patient-centred outcomes, which our pooled data suggest are promising.

## Background

Neurogenic bladder (NGB) is characterized by reduced bladder capacity and/or decreased compliance, frequently accompanied by bladder trabeculations and high-pressure voiding. This condition may arise from either congenital or acquired factors, leading to urinary incontinence, vesicoureteral reflux (VUR), and, if left untreated, progression to renal function impairment. Approximately 50% of pediatric patients with NGB achieve urinary continence (1). The primary management objectives for NGB are to prevent renal deterioration and to enhance the quality of life.

Non-surgical interventions for NGB include clean intermittent catheterization (CIC), anticholinergic medications, medical devices, and neuromodulation; however, more than 60% of patients eventually require surgical intervention (1). Surgical options, such as Mitrofanoff appendicovesicostomy (MAPV), bladder augmentation, and urinary diversion, are often necessary to preserve urinary function.

Open augmentation ileocystoplasty (OAI) is associated with increased bladder capacity, reduced intravesical pressure, enhanced compliance, and diminished VUR, while also maintaining satisfactory continence and providing long-term renal protection (2). Nevertheless, OAI is often accompanied by substantial trauma and postoperative complications (3).

Minimally invasive bladder reconstruction techniques encompass: (A) laparoscopic and robotic-assisted augmentation cystoplasty; (B) laparoscopic or robotic Mitrofanoff procedures for the creation of a catheterizable channel; (C) combined bladder augmentation with a catheterizable channel for continent cutaneous ileostomy; and (D) robotic implantation of an artificial urinary sphincter at the bladder neck (4). The first report of complete laparoscopic augmentation ileocystoplasty (LAI) emerged in 2002, demonstrating it to be a safe and efficacious minimally invasive approach, with clinical outcomes comparable to those of the OAI procedure (5, 6).

The rapid advancement of robotic surgical technology has generated significant interest in robotic-assisted laparoscopic procedures for complex urinary reconstruction, primarily due to their flexibility. Robot-assisted laparoscopic augmentation ileocystoplasty (RALAI) was first introduced for patients with NGB in 2008 (7, 8). However, the efficacy and safety of the RALAI procedure remain inadequately understood.

## Methods and patients

### Case presentation

We present a case involving an 8.7-year-old boy with a 7-year history of urinary incontinence that was unresponsive to anticholinergic agents and CIC, significantly impacting his social interactions and quality of life. There was no family history of urinary disorders. Preoperative renal ultrasound showed severe hydronephrosis, reduced renal parenchymal echo, and thinning of cortical. Magnetic resonance imaging (MRI) revealed occult spina bifida at the S1/S2 level. Urodynamic studies indicated low bladder capacity (198 ml), poor compliance (13.6 ml/cm H_2_O), elevated maximum detrusor pressure (24 cm H_2_O), high post-void residual urine volume (250 ml), and a reduced flow rate (2.32 ml/s). A voiding cystourethrogram (VCUG) demonstrated NGB with grade I left VUR (Figures 1, 2).

**Figure 1:**
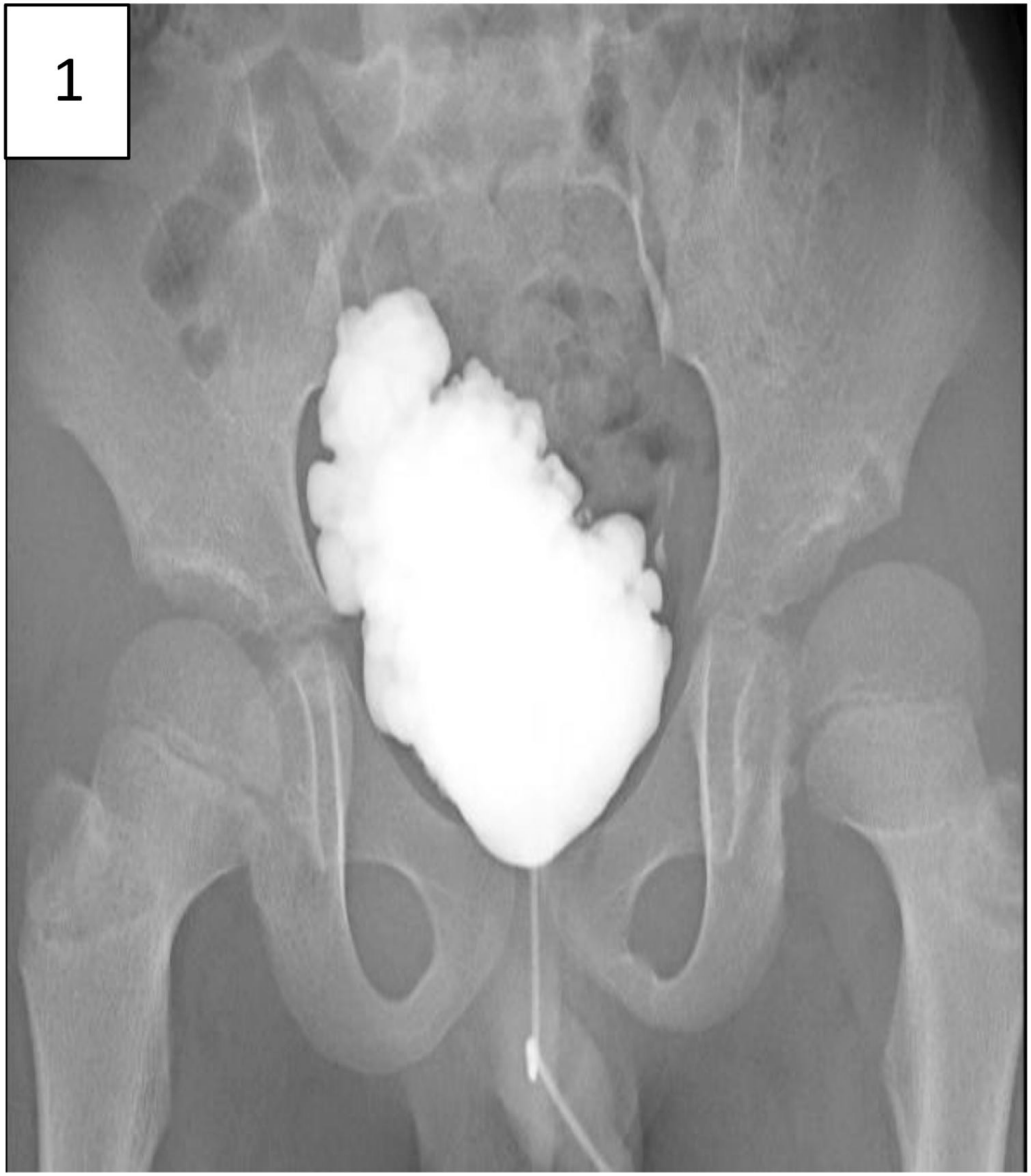
VCUG illustrates bladder trabeculations, diverticula, and reflux into the left distal ureter.

**Figure 2:**
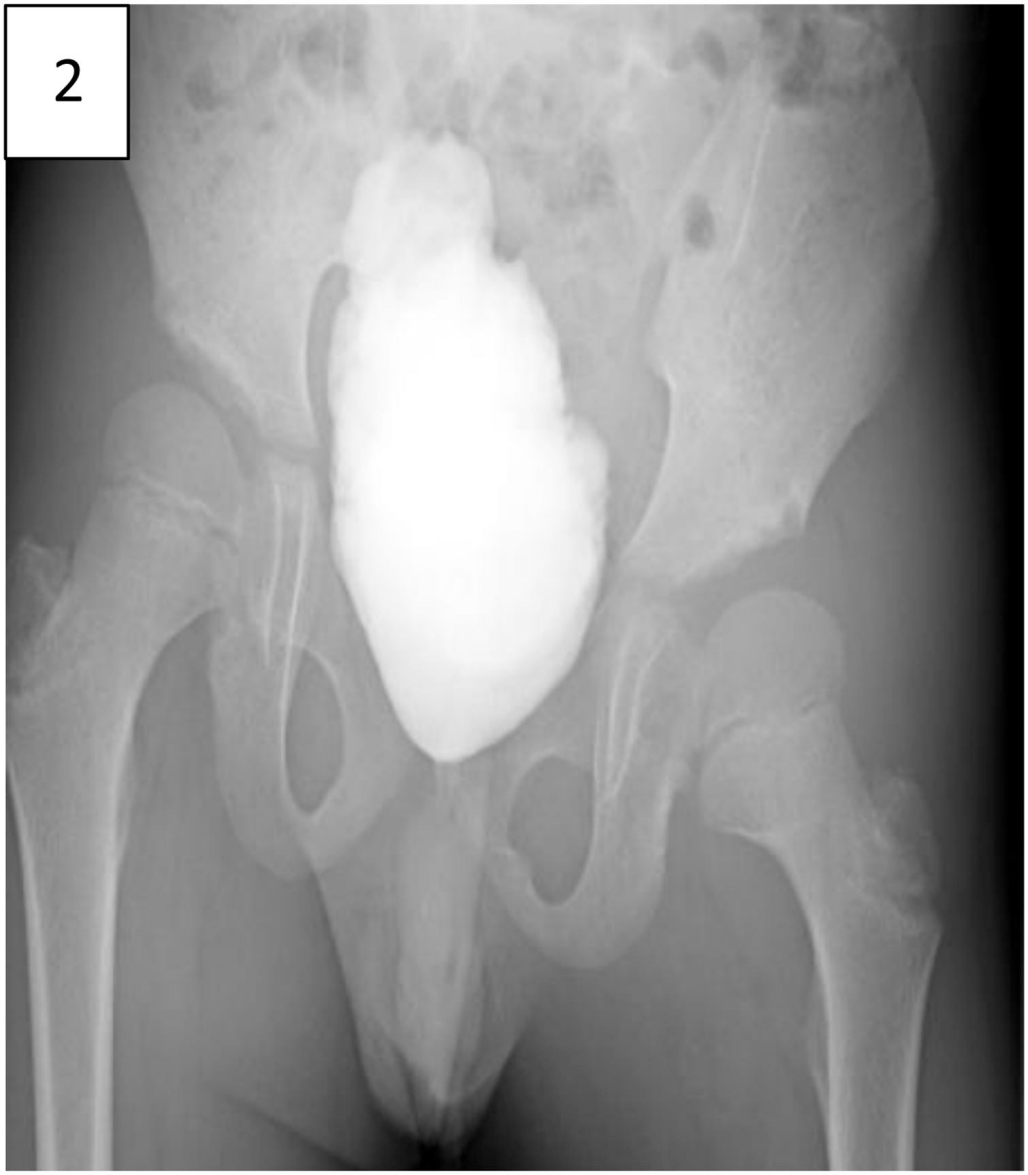
VCUG demonstrates significant contrast retention in the bladder post-voiding.

### Surgical technique

The RALAI procedure was executed using a standardized technique with the da Vinci Surgical System (Intuitive Surgical, Sunnyvale, CA, USA) configured in a four-arm setup. Perioperative management adhered to our institution's pediatric protocols. Intravenous antibiotic prophylaxis was administered prior to the incision and continued throughout the immediate postoperative period. Following the induction of general anesthesia, the patient was positioned in a supine 30° Trendelenburg position. An 8-Fr Foley catheter was inserted, and a trans-umbilical incision was made for the laparoscopic trocar, with additional working ports established as documented in the literature (9).

A pneumoperitoneum was established at 10 mmHg to facilitate the insertion of the robotic arm. The appendix was identified, mobilized, ligated, and transected 0.5 cm from its base. A 10-Fr catheter was inserted into the appendix, directing it towards the right bladder (Figure 3A). The peritoneum overlying the bladder was incised, the urachus was transected, and a 4 cm incision was made in the right bladder wall. The tip of the appendix was sutured to the bladder, and the muscle layer was closed using 4-0 Vicryl sutures (Figure 3B). A 25 cm segment of the proximal ileum was selected for bladder augmentation, with its mesenteric vessels ligated and the segment transected. An anastomosis was then performed between the proximal ileum and the ends of the ileum (Figure 3C), and the mesentery was sutured closed. A midline incision was made in the bladder, extending to the bladder neck, which revealed thickened walls, mucosal trabeculations, and reduced capacity. Following irrigation and detubularization of the ileal segment, a detubularized ileal patch was constructed and affixed to the bladder using 3-0 inverted sutures (Figure 3D). A 1.0 cm incision was made on the right abdominal wall to create a stoma from the distal appendix, and a 10-Fr ureteral stent was inserted to support the appendiceal conduit. The surgical area was irrigated, a pelvic drain was placed, and the incisions were closed.

**Figure 3:**
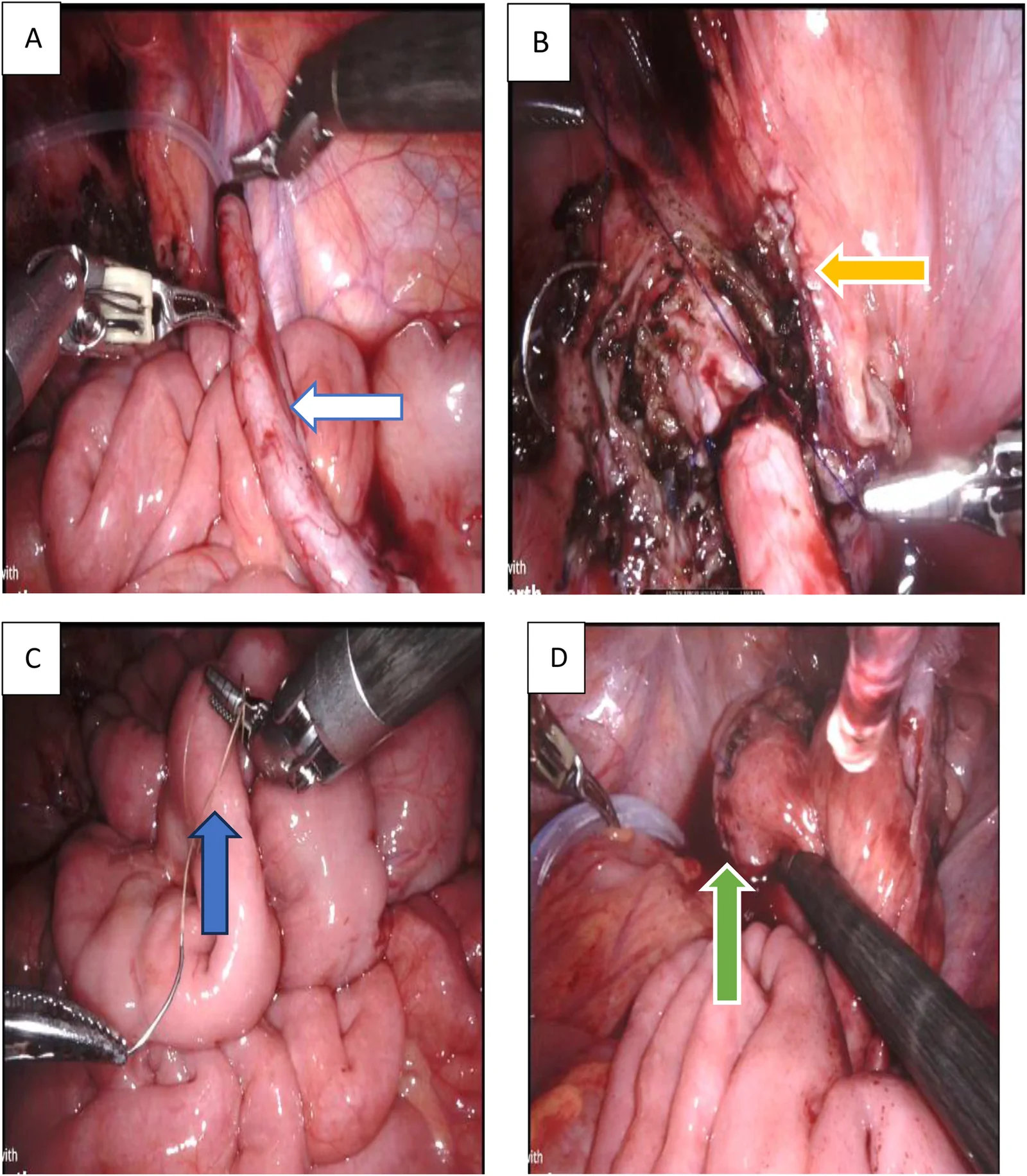
**(A)** Robotic-assisted laparoscopic mitrofanoff appendicovesicostomy (white arrow). **(B)** Embedding the distal appendix in bladder mucosa (Yellow arrow). **(C)** Using an ileal segment for bladder augmentation (Blue arrow). **(D)** Reconstructing bladder and bowel continuity (Green arrow).

The procedure lasted 605 min, with an estimated blood loss less than 100 ml, and the patient remained stable without complications. By the third postoperative day, the stoma was clean with intact skin. Ten days after the stent removal, an 8-Fr silicone catheter was inserted through the stoma for urinary drainage, with an average output of 150–200 ml every 2–3 h, and was removed on the thirteenth day. Intermittent bladder irrigation with saline was employed.

At the 3-year follow-up, the patient did not undergo formal postoperative urodynamic study as the patient and family declined invasive testing; thus, functional improvement was inferred from imaging and clinical outcomes. Ultrasound revealed a 92% increase in bladder capacity, from 198 ml to 380 ml, and clinically, there were no urinary tract infections (UTIs), bladder stones, or stomal complications. The patient had resumed intermittent catheterization and spontaneous voiding. Postoperatively, the patient discontinued the use of diapers, resulting in enhanced quality of life and improved social interactions. “Since the operation, he no longer requires diapers, attends school and social activities with increased confidence, and the overall quality of life for our family has significantly improved.”

### Literature review

A comprehensive literature review was conducted for the period from January 1, 2004, to December 31, 2024, utilizing databases such as PubMed, Web of Science, CNKI, and Wanfang. The search employed the keywords “neurogenic bladder,” “ileocystoplasty,” and “robot,” with the following search string: (“Neurogenic Bladder”[MeSH] OR “neurogenic bladder”[tiab] OR “neurogenic bladder dysfunction”[tiab]) AND (“Augmentation Cystoplasty”[MeSH] OR “augmentation cystoplasty”[tiab] OR “augmentation ileocystoplasty”[tiab] OR “ileocystoplasty”[tiab] OR “bladder augmentation”[tiab]) AND (“Robotic Surgical Procedures”[MeSH] OR “robotic”[tiab] OR “robot-assisted”[tiab] OR “robotic-assisted”[tiab] OR “robot assisted”[tiab] OR “da Vinci”[tiab]) AND (“ileum”[MeSH] OR ileal[tiab] OR ileum[tiab] OR ileocystoplasty[tiab]). During the screening phase, the language of publications was restricted to English and Chinese. This review was conducted as a structured literature review with descriptive pooled analysis, rather than a formal systematic review or meta-analysis. Accordingly, a protocol was not registered with PROSPERO.

The search results were initially screened with a focus on case reports and original research articles, selecting studies based on their titles and abstracts. The inclusion criteria were as follows: (1) a diagnosis of NGB that is refractory to conservative management and/or presents urodynamic evidence of low capacity, poor compliance, or high storage pressures that pose a risk to the upper urinary tracts; (2) the patient underwent RALAI utilizing the ileum as the bowel segment. Exclusion criteria encompassed procedures performed via open or conventional laparoscopic (non-robotic) approaches, studies concerning non-neurogenic disorders, augmentations using non-ileal segments, and duplicate data from the same institution.

We evaluated the methodological quality and risk of bias of the included studies utilizing the Joanna Briggs Institute critical appraisal tools appropriate for each study design. Each study underwent independent appraisal by two reviewers, with any disagreements resolved through discussion or, if necessary, adjudication by a third reviewer. To prevent the double counting of patients, we screened the included reports for potential duplicate populations during data extraction. This potential overlap was assessed by comparing institutions and departments, recruitment/enrollment periods, corresponding or overlapping author lists, reported sample sizes and baseline characteristics, as well as any textual clues indicating cohort identity. To avoid double-counting, when multiple reports from the same institution with overlapping authorship and timeframes were identified, we retained only the most recent and largest series Adamic et al. (14) and excluded the earlier report from the same group. A PRISMA flow diagram (Figure 4) was employed to present the search results.

**Figure 4:**
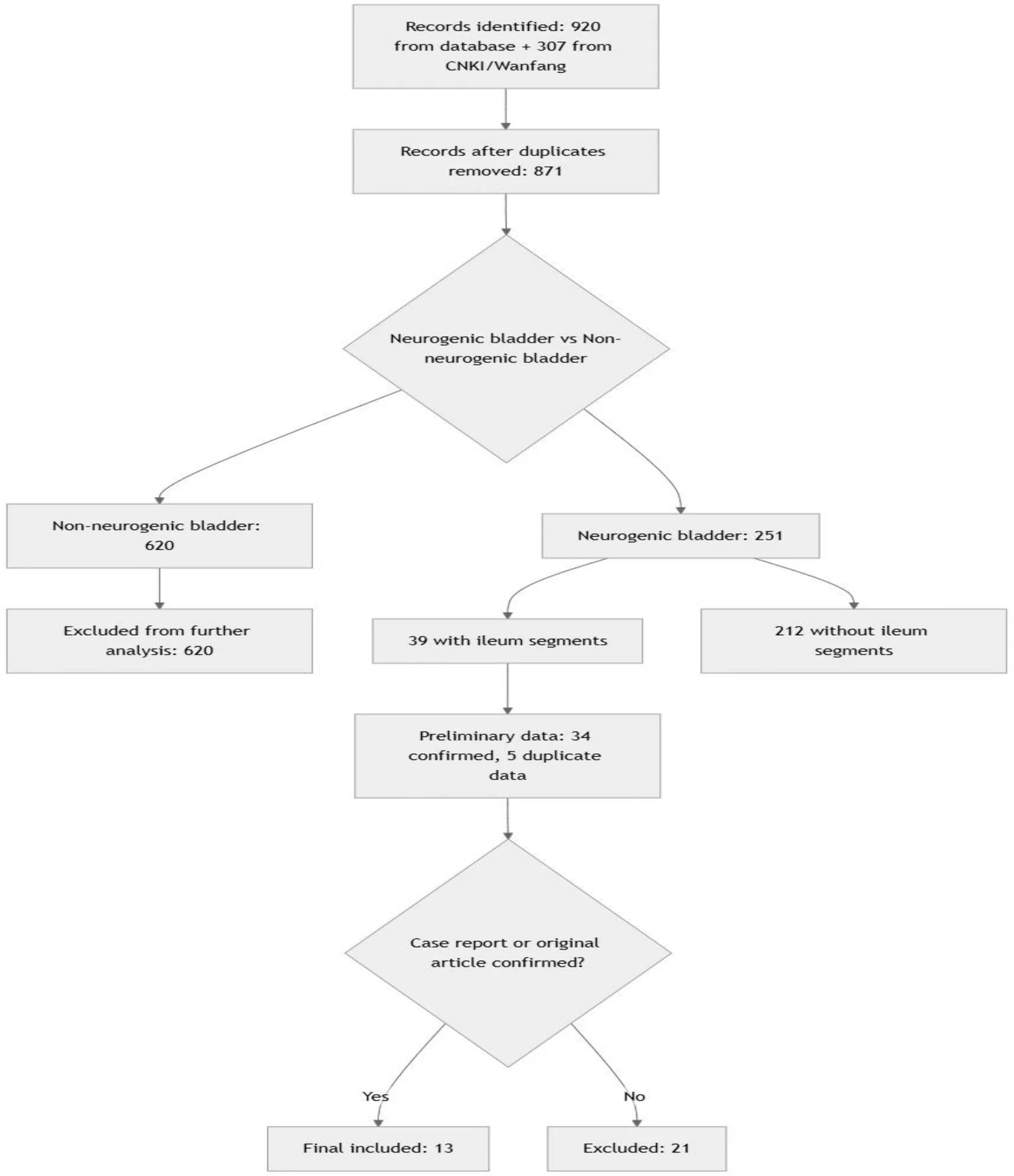
Flow chart showing the results of the literature search.

The primary outcome was procedural success, defined as the completion of the RALAI procedure without unplanned conversion to an open approach, which we explicitly considered as a process measure. Intraoperative complications defined as adverse events directly attributable to surgical technique that cause tissue injury or hemorrhage, and that are potentially preventable with improved skill or technique, like visceral injury, major vascular injury, or anesthetic events. Intraoperative adverse events defined as unplanned intraoperative occurrences that affect the procedure but do not necessarily reflect surgical error; includes conversions and device-related issues. For secondary clinical outcomes, we predefined early complications as events occurring within 90 days postoperatively. These included urinary continence, intestinal obstruction, anastomotic dehiscence and leakage, perforation, surgical site infections, and UTIs. Given the heterogeneous designs of the included studies (case reports and small case series), we performed a descriptive pooled analysis rather than a formal meta-analysis. Crude proportions and their 95% confidence intervals (Wilson score) are reported for key outcomes. In accordance with ethical standards, written informed consent was obtained from the parents of the case for the publication of identifiable images or data. The study received approval from the Ethics Committee of Wuhan Children's Hospital (2021R030-E01).

## Results

Our index case is presented in detail in the narrative above and is also summarized in the bottom row of Table 1 (**bolded for clarity**), allowing for a direct side-by-side comparison of its preoperative and postoperative parameters against the pooled cohort from the literature. Following a comprehensive review, 13 articles were selected based on predefined inclusion criteria, encompassing a total of 92 cases, including the case reported herein. Among the 14 included studies, 4 were assessed as high quality, 9 as moderate quality, and none as low quality. The clinical data reviewed encompassed preoperative examinations, intraoperative conditions, and postoperative follow-up at 90 days. Key outcomes assessed included the success rate of the robotic-assisted laparoscopic approach for urinary reconstruction, intraoperative adverse events (including conversions and device malfunctions), postoperative complications, and the necessity for secondary surgical revisions. Demographic, treatment, and outcome data are documented and summarized in Tables 1, 2.

**Table 1:** RALAI management of NGB—demographical characteristics and outcomes.

Reference	*N*	Age	Operation time (mins)	Concomitant procedure	Intraoperative adverse events	Conversion	Restore bowel continuity	Estimated blood loss (ml)	Preoperative capacity (ml)	Postoperative capacity (ml)	Early postoperative complications	Secondary surgery revision
Yeow et al. (10)	1	37	180	None	None	Not require	Stapler	Minimal blood loss	90	160	None	Not require
Ramos et al. (11)	1	12	380	None	None	Not require	Anastomosis	Less than 100	167	N/A	None	Not require
Zhu et al. (12)	12	Mean 38 (12–67)	108–132	None	None	Not require	Anastomosis	84.00 ± 23.20	95.67 ± 39.10	435.83 ± 33.56	None	Not require
Grilo et al. (13)	10	Median 44 (28–58)	Median 250 (210–268)	None	None	Not require	Stapler	75 (50–205)	260 (135–380)	515 (415–615)	Two UTIs, two paralytic SBO, and one urinary fistula	Not require
Adamic et al. (14)	24	Median 11.7	Mean 573	MAPV 16, ACE 8, BNC 6	4	Two patients with kyphosis and two with dense adhesions	Anastomosis	103 (64–142)	214 (106–322)	437 (380–494)	Two SBO, five UTIs, one SSI, one bladder neck dehiscence, one bladder perforation, and four difficulty catheterizing	One bladder neck dehiscence required BNC, four difficulty catheterizati-on required Mitrofanoffscopy
Halleran et al. (15)	7	Mean6.4 (3.8–10.1), median 5.4	mean 526 (313–724)	Malone 7, MAPV 6, BNC 5	None	Not require	Anastomosis	Median 25 (10–100)	N/A	N/A	One Mitrofanoff stoma stenosis and one colo-colonic anastomotic fistula	The fistula required surgical repair
Kibar et al. (16)	2	Mean 11.5(11–12)	340 (320–360)	None	None	Not require	Anastomosis	325 (225∼400)	165 (150–180)	340–350	None	Not require
Caputo et al. (17)	1	35	286	None	None	Not require	Stapler	50	96	165	None	Not require
Wiestma et al. (18)	1	6	496	None	None	Not require	Anastomosis	N/A	N/A	N/A	None	Not require
Flum et al. (19)	22	Median 30 (19–60)	Mean 365 (220–788)	4 Monti, 3 MAPV	1	Creation of the appendicovesicostomy with tension	Stapler	Mean 110 (30–250)	Mean 355 (160–601)	488 (251–648)	Three SBO, two bladder perforation, one anastomotic disruption, and one sepsis from UTI	One bladder perforation,one ileovesicostomy-augment anastomosis disruption and one SBO perforation require reoperation
Rey et al. (20)	4	Median 32 (22––65)	225–270	MAPV or double Yang-Monti ileal conduit	None	Not require	Anastomosis	140 (50–300)	N/A	N/A	Retrovesical abscess in two patients, and four patients developed UTIs	Two retrovesical abscess need surgical drainage
Gould et al. (21)	5	Mean 43 (24–55)	384 (354–438).	double Yang-Monti ileal conduit	1	Robotic malfunction	Stapler	Mean 180 (100–250)	110 (70–220)	N/A	One SBO, one UTI, and one stoma incontinence	One stoma incontinence require revision
Kang et al. (22)	1	54	300	None	None	Not require	Anastomosis	225	100	350	None	Not require
**You et al.**	**1**	**8.7**	**605**	**MAPV**	**None**	**Not require**	**Anastomosis**	**Less than 100**	**198**	**380**	**None**	**Not require**

**Table 2:** Summarized complications.

First author	N	Conversion	Complication	Stomal stenosis	Stomal incontinence	SBO	Dehiscence/leakage	SSI	UTI	Second surgery
Yeow et al.	1	–	–	–	–	–	–	–	–	–
Ramos et al.	1	–	–	–	–	–	–	–	–	–
Zhu et al.	12	–	–	–	–	–	–	–	–	–
Grilo et al.	10	–	5	–	–	2	1	–	2	–
Adamic et al.	24	4	14	4	–	2	2	1	5	5
Halleran et al.	7	–	2	1	–	–	1	–	–	1
Kibar et al.	2	–	–	–	–	–	–	–	–	–
Caputo et al.	1	–	–	–	–	–	–	–	–	–
Wiestma et al.	1	–	–	–	–	–	–	–	–	–
Flum et al.	22	1	7	–	–	3	3	–	1	3
Reyet al.	4	–	4	–	–	–	–	2	4	2
Gould et al.	5	1	3	–	1	1	–	–	1	1
Kang et al.	1	–	–	–	–	–	–	–	–	–
**You et al.**	**1**	–	–	–	–	–	–	–	–	–
Total	92	6 (6.5%)	35 (38.0%)	5 (5.4%)	1 (1.1%)	8 (8.7%)	7 (7.6%)	3 (3.3%)	13 (14.1%)	12 (13.0%)

MAPV, Mitrofanoff appendicovesicostomy; ACE, antegrade continence enema; BNC, bladder neck closure; SBO, small bowel obstruction; SSI, surgical site infection; UTI, urinary tract infection; N/A, not available.

In our study, the median operative duration for the RALAI procedure was 340 min, no intraoperative complications were observed. A majority of patients demonstrated a significant enhancement in postoperative bladder capacity. As a measure of procedural feasibility, the RALAI procedure was completed without unplanned conversion in 93.5% of cases. The conversion rate of 6.5% reflects the technical demands and patient-selection challenges inherent to this complex reconstruction. Six cases (6.5%) necessitated conversion to open surgery, and one incident of robotic device electrical failure was documented; these instances are thus classified as intraoperative adverse events. The reasons for conversion included limited intra-abdominal working space in two patients with kyphosis, dense adhesions and unfavorable appendiceal anatomy in two patients, and insufficient appendiceal length or tension in one patient. Apart from the conversions and the single device malfunction, no intraoperative hemorrhages, visceral injuries, or anesthetic complications were recorded within the cohort.

In the early postoperative period, complications were observed in 35 patients (38.0%), including one instance of Mitrofanoff stomal stenosis, seven cases of small bowel obstruction, 13 instances of UTIs, one case of surgical site infection, and one case of urinary fistula, all of which were managed conservatively. Surgical revision was necessary for 12 patients (13.0%), including one case of bowel obstruction that progressed to perforation, four cases of stomal stenosis requiring Mitrofanoffscopy, two instances of retrovesical abscess necessitating surgical drainage, one case of stomal incontinence, and four cases of dehiscence, leakage, or perforation. No patients required bladder re-augmentation. Concurrent MAPV procedure was explicitly reported in 25 of 92 patients (27.2%, 95% CI 18.6–37.5%) across the included studies. In the remaining studies where the performance of MAPV was not mentioned, we conservatively assumed that no MAPV procedure was performed. It is possible that some of these unreported cases also underwent MAPV, in which case the true proportion would be higher than 27.2%; however, the available data do not allow us to confirm this.

## Discussion

Augmentation cystoplasty is considered a second-line treatment for NGB, and ileum is the most commonly used gastrointestinal segment in the procedure (1, 2). Augmentation ileocystoplasty plays a crucial role in safeguarding the upper urinary tracts in NGB patients who do not respond to non-surgical treatments (1, 4). Beyond the ileocystoplasty technique that is the focus of our study, several other surgical strategies and technical alternatives exist for managing NGB refractory to conservative treatment, as comprehensively reviewed by Barone et al. (23) in the context of urinary diversion evolution. Bladder auto-augmentation offers a non-enteric alternative that avoids gastrointestinal segment-related metabolic complications, though its long-term efficacy is less well established compared to enterocystoplasty. When enteric augmentation is indicated, the choice of bowel segment—ileum, cecum, sigmoid colon, or even stomach—carries different metabolic and functional implications; ileum is most favored due to its favorable distensibility, capacity, and long-term mucosal atrophy that reduces electrolyte exchange.

LAI procedure was initially documented by Gill et al. (24) in 2,000, with the bowel patch and bowel anastomosis performed extracorporeally. Subsequently, Elliott et al. (5) and Meng et al. (25) reported the first instances of entirely intracorporeal LAI in 2002. The introduction of RALAI in the pediatric population was first reported in 2008 (7). In 2010, Gundeti et al. (26) conducted a study involving six pediatric patients with NGB who underwent completely intracorporeal RALAI, with or without simultaneous MAPV. The study concluded that the procedure was safe, effective, and feasible, providing potential advantages such as reduced recovery time and enhanced cosmetic outcomes.

In this study, the RALAI procedure demonstrated a completion rate of 93.5%. The median duration of surgery was 340 min, substantially longer than the 207 min reported for traditional methods, attributable to the complexity and learning curve associated with the RALAI technique (9). As advancements in robotic technology for bladder reconstruction continue, enhanced surgical techniques, such as the use of endoscopic staplers for bowel continuity reconstruction (8), are anticipated to decrease operative time. The operative time in our index case (605 min) was at the upper end of the range reported in the literature (median 340 min, range 108–788 min). Several factors contributed to this prolonged duration. First, this was the first RALAI case performed at our institution, and the procedure inevitably involved a steep learning curve, including the acquisition of proficiency in intracorporeal suturing, bowel handling, and robotic port placement. Second, the procedure was a complex combined operation, incorporating both augmentation ileocystoplasty and a concomitant MAPV, which substantially adds to the operative time compared with augmentation alone. Third, we employed a completely intracorporeal approach for all steps, including bowel resection, detubularization, and hand-sewn bowel anastomosis, which, while offering the advantages of minimal access, requires more time than extracorporeal alternatives. Notably, no intraoperative complications were observed during RALAI procedures, and there was a significant increase in postoperative bladder capacity, approaching physiological levels, the outcomes of our case (92% capacity increase, no complications) align with the favorable trend observed in the majority of the pooled RALAI series. Comparative studies have revealed no significant difference in median postoperative bladder capacity between this method and the OAI technique (9).

Among our cohort, 6 out of 92 cases (6.5%) necessitated conversion to open surgery due to challenges such as limited intra-abdominal space resulting from kyphosis, dense adhesions, appendiceal abnormalities, and robotic electrical failure. These findings underscore the importance of meticulous patient selection, particularly for individuals with scoliosis, prior abdominal surgeries, or atypical appendiceal anatomy, highlighting the critical role of preoperative CT/MRI assessments. While the conversion rate is a useful measure of procedural feasibility, the ultimate value of RALAI for patients lies in its ability to improve continence, protect renal function, and reduce the burden of UTIs and reoperations.

In our study, 35 patients (38.0% of the cohort) experienced complications, which is a lower incidence compared to the 50% to 62% reported in historical OAI cohorts (9, 27). The most prevalent postoperative complications were UTIs, occurring in 14.1% of patients with NGB, consistent with rates observed in the open approach (2). Although the majority of patients were managed conservatively, 12 patients (13.0% of the cohort) required a secondary surgical revision due to complications such as anastomotic rupture, leakage, perforation, stomal stenosis, or incontinence. The incidence of reoperation was significantly lower than that associated with the OAI approach (2). Robotic-assisted laparoscopic procedures in complex urinary reconstruction confer advantages such as precise dissection and enhanced suturing, which contribute to a reduction in complications such as stomal stenosis, necrosis, bowel obstructions, and anastomotic dehiscence or leakage (12, 14). Experiments utilizing porcine models have demonstrated that the robot-assisted laparoscopic approach results in less tissue damage, thereby reducing intestinal adhesions and complexity, and consequently lowering the risk of complications (28).

Our study primarily captured early postoperative complications, as this was the most consistently reported timeframe across the included case series. Long-term outcomes—including bladder stone formation, metabolic disturbances, chronic bacteriuria, and the eventual need for re-augmentation—remain critically important but are insufficiently documented in the current RALAI literature. 5.2–13.4% of patients who underwent OAI procedure required re-augmentation (29). In our pooled cohort, no patient required re-augmentation during the available follow-up periods, and our own index case remained stone-free at 3 years. We therefore recommend that future studies adopt standardized, extended follow-up protocols to capture these delayed complications, which may ultimately determine the true comparative effectiveness of RALAI.

In the study, 25 out of 92 patients (27.2%) underwent simultaneous MAPV during the initial procedure. The use of an appendiceal conduit for intermittent catheterization is advantageous as it facilitates convenience, reduces bladder pressure, and may enhance voluntary urination. Nevertheless, there is a lack of consensus regarding the simultaneous performance of these procedures, given that additional surgical interventions can prolong operative time. The MAPV procedure is widely regarded as a reliable and preferred technique for establishing a continent urinary stoma in patients with NGB (30). However, the long-term outcomes of isolated bladder outlet procedures for neurogenic incontinence are frequently unsatisfactory (31). Another study suggests that managing NGB without augmentation cystoplasty is safe and effective only for a subset of NGB patients, even among patients with normal preoperative bladder compliance and capacity, approximately 40% still require augmentation cystoplasty (32).

Famakinwa et al. (33) and Wille et al. (34) contend that there is no direct correlation between augmentation cystoplasty combined with MAPV and patient outcomes. Outcomes associated with MAPV, regardless of the inclusion of ileocystoplasty, appear promising. A retrospective cohort study (35) examined the influence of detrusor leak point pressure and bladder trabeculations on surgical strategies for incontinent pediatric patients with NGB. The study concluded that the combination of augmentation cystoplasty and the Mitrofanoff procedure improves outcomes for patients with elevated detrusor leak point pressure and significant bladder trabeculations. Over a three-year follow-up period, no patients experienced deterioration of the upper urinary tract, suggesting that these surgical interventions are particularly effective in specific clinical contexts.

Due to the potential for increased operative complexity, duration, and risk of adverse events associated with concomitant MAPV, the pooled complication and reoperation rates may be confounded by these combined procedures. As a result, the pooled complication rates presented in this study represent a heterogeneous population (both with and without concomitant MAPV). Further research is warranted to evaluate the benefits of this combined approach.

### Limitations

Initially, we considered procedural success, defined as completion without conversion, as a valuable metric for assessing feasibility and operative performance. However, it is important to recognize that this is a process measure and does not directly translate to patient benefit. Clinical outcomes such as continence, independence from CIC, and the incidence of UTIs are more pertinent to patient quality of life. Our index case lacks formal postoperative urodynamic data at long-term follow-up, which precludes a definitive assessment of the achieved compliance and pressure reduction.

Secondly, the majority of the studies included were small case series or reports, which are limited by potential selection bias, incomplete outcome reporting, and short-term follow-up. These limitations diminish the reliability of the pooled complication rates.

Additionally, the results amalgamate data from both pediatric and adult populations and combine standalone RALAI with cases involving concomitant procedures such as MAPV, bladder neck closure (BNC), and antegrade continence enema (ACE). These factors are likely to affect complication and re-operation rates. Therefore, future research should emphasize the standardized reporting of patient-centered endpoints and ensure adequate follow-up. It should also specify subgroup analyses based on age group and the presence of concomitant procedures, and, where feasible, compare stapled vs. hand-sewn bowel continuity.

Lastly, we acknowledge the potential for publication bias inherent to this type of aggregated case-series evidence. Studies reporting positive or uneventful outcomes with robotic-assisted procedures are more likely to be published than those describing technical failures, conversions, or complex complications. This selective reporting may result in an overestimation of the procedural success rate and an underestimation of adverse event rates in our pooled analysis. We strongly encourage the establishment of prospective, multi-institutional registries to provide more unbiased estimates of RALAI outcomes.

## Conclusions

Based on our pooled analysis of 92 patients, we conclude that RALAI is a technically feasible minimally invasive option for carefully selected patients with NGB, with a completion rate without conversion of 93.5% and an overall conversion rate of 6.5%. However, the clinical value of the procedure should be assessed primarily through patient-centred outcomes, which in our data demonstrate substantial increases in bladder capacity, preservation of upper urinary tracts, an early complication rate of 38.0%, and a reoperation rate of 13.0%. Patient selection remains critical, as kyphosis, and unfavorable appendiceal anatomy were key predictors of conversion. We acknowledge that the heterogeneity of the included studies—mixing pediatric and adult populations, and combining pure RALAI with cases involving concomitant MAPV—limits the robustness of our pooled estimates. Future prospective, multi-institutional registries with extended follow-up and standardized patient-reported outcomes are urgently needed to compare its long-term effectiveness, durability, and cost-effectiveness against open and other minimally invasive approaches.

## Data Availability

The original contributions presented in the study are included in the article/Supplementary Material, further inquiries can be directed to the corresponding author/s.
